# Change in Quality of Life after Rehabilitation in Cambodia among Adults with Lower Limb Amputation

**DOI:** 10.1298/ptr.R0039

**Published:** 2026-06-30

**Authors:** Sit SONG, Phearavin PHENG

**Affiliations:** 1 School of Public Health, National Institute of Public Health, Cambodia; 2 The Public Health Doctoral School (EDSP), Paris-Saclay University, France

**Keywords:** Rehabilitation, Quality of life, Disability, Amputation

## Abstract

**Objectives:**

This study aimed to describe the quality of life (QoL) of persons with lower limb amputations (LLAs) in Cambodia both before and after rehabilitation, and to identify predictor variables associated with increased QoL outcomes post-rehabilitation.

**Methods:**

A total of 164 participants with LLA from 7 physical rehabilitation centers (PRCs) across Cambodia completed the baseline assessment; however, only 125 participants (58% below- or through-knee amputations; 98% unilateral) completed the 3-month follow-up, with 39 participants lost to follow-up. Participants were interviewed using the Comprehensive Quality of Life Scale Adult questionnaire (which includes both objective and subjective scores). Baseline data were collected on the first day of admission at the PRC, following referrals from hospitals after the limb surgery. Follow-up data were gathered 3 months after discharge from rehabilitation. A paired t-test was used to assess the difference in QoL scores, and linear regression identified factors potentially associated with changes in QoL.

**Results:**

Our findings indicate that both objective and subjective QoL scores of persons with LLA increased significantly 3 months after rehabilitation (p <0.001). The absence of residual stump pain was significantly associated with higher QoL scores (p = 0.002). In contrast, a longer delay (>12 months) between amputation and receipt of rehabilitation services was associated with a smaller improvement in subjective QoL (p = 0.023).

**Conclusions:**

The findings suggest that early rehabilitation after amputation and the inclusion of effective pain management may be associated with better QoL outcomes; however, these approaches should be evaluated through further research to validate their impact.

## Introduction

Limb amputation leads to permanent disability and impacts significantly on a person’s life and function. Compared to the general population, persons with lower limb amputations (LLAs) have a poorer quality of life (QoL)^1)^. Globally, the incidence of LLA in 2011 ranged from 5.8 to 31 per 100000 in the total population^2)^. In 1996, due to the civil wars, Cambodia had a high incidence of amputations (mostly LLA), up to 390.63 per 100000 per year, compared to 44 per 100000 in Vietnam^3)^. By 2025, Cambodia recorded 9101 persons with amputations caused by landmines and unexploded ordnance (UXO), accounting for 14% of the total 65125 casualties^4)^. Since 1992, Cambodia has established 11 Physical Rehabilitation Centres (PRCs)^5)^. Between 2012 and 2017, these centers served an average of 27160 persons with disabilities annually, of whom 3723 (35%) were persons with amputations^6)^.

In Cambodia, most amputations seen in PRCs are due to mines or UXO, or road accidents^6,7)^, in contrast to developed countries, where the majority of amputations are caused by peripheral vascular disease. In recent years, the number of amputations caused by mines and UXO in Cambodia has gradually decreased, while those resulting from road accidents have increased alongside the volume of road traffic, with an average of more than 9 severe injuries per day in 2023^6,7)^.

Rehabilitation plays a crucial role in improving the QoL for persons with LLA by addressing its various physical, psychological, and social aspects^2,8)^.

It has been demonstrated that rehabilitation improves the mobility and independence of persons with LLA, particularly those with transtibial amputation^9)^.

Several instruments have been developed to assess QoL among persons with LLA, including the Trinity Amputation and Prosthesis Experience Scales (TAPES), the World Health Organization Quality of Life-Brief Version (WHOQOL-BREF), and the Comprehensive Quality of Life Scale-Adult Version 5 (ComQOL-A5)^10,11)^. The ComQOL-A5 is sensitive enough to detect changes in QoL over time, making it essential for longitudinal studies evaluating the effectiveness of interventions such as rehabilitation^10,12)^.

Several factors have been identified as influencing or predicting the impact on QoL for persons with LLA after rehabilitation. These include the absence of stump pain, which is significantly associated with an increased QoL score, absence of phantom limb pain, a shorter waiting time (e.g., <6 months) between amputation and receiving rehabilitation services, receiving support (motivational or financial) from relatives or friends before rehabilitation, ability to walk with a prosthesis, and prosthesis satisfaction^13,14)^. Conversely, factors negatively associated with QoL include older age, being male, longer time since amputation, and presence of diabetes^15)^.

Cross-sectional studies in Cambodia^16)^ and elsewhere^1,13,14)^ have shown that receiving rehabilitation services is associated with a better QoL for persons with amputation. However, there have been no longitudinal studies in Cambodia and few elsewhere^1,14)^ that have looked at changes in QoL following rehabilitation among adults with LLA. This gap in evidence from longitudinal studies provides the rationale for the hypothesis and objectives of the study, which aims to systematically measure QoL changes for persons with LLA after rehabilitation compared to before rehabilitation, and identify key predictors influencing these outcomes.

In Cambodia, PRCs currently operate outside of the formal health system, as they are managed by the Ministry of Social Affairs, Veterans and Youth Rehabilitation^5,6)^. Persons undergoing amputation surgery at hospitals are typically discharged and then referred to PRCs for subsequent rehabilitation services^5,6)^.

The hypothesis for this prospective study was that the QoL scores of persons with LLA would increase after rehabilitation intervention. This hypothesis directly addresses the identified research gap regarding longitudinal evidence on QoL changes post-rehabilitation. The objectives of the study were to describe the QoL for persons with LLA in Cambodia before and after rehabilitation and to investigate which predictor variables were associated with increased QoL scores for persons with LLA after rehabilitation.

## Methods

### Setting

The study was conducted at 7 PRCs in Cambodia across 5 provinces and the capital city.

### Study design

This was a prospective cohort study that sought to compare the QoL scores of persons with LLA before and after receiving rehabilitation services.

### Study population and selection criteria

One hundred and sixty-four persons with LLA aged 18 years or older admitted to any of the 7 PRCs between September 2013 and May 2015 who satisfied the eligibility criteria were recruited to join the study. The exclusion criteria were refusal to provide informed consent, previous rehabilitation at any of these 7 PRCs, multiple physical disabilities, severe or chronic medical conditions (such as stroke, severe heart failure, and tuberculosis), or a history of severe or chronic psychological disorder. The selection criteria were confirmed by the physical therapists, who assessed participants for rehabilitation services at the 7 PRCs. Of the 164 persons with LLA originally recruited, 39 were subsequently excluded as they were unable to be contacted to complete the follow-up questionnaire 3 months after discharge from rehabilitation services. A total of 125 persons therefore fully participated in the study by completing the pre- and post-rehabilitation questionnaires (Fig. 1).

**Fig. 1.**
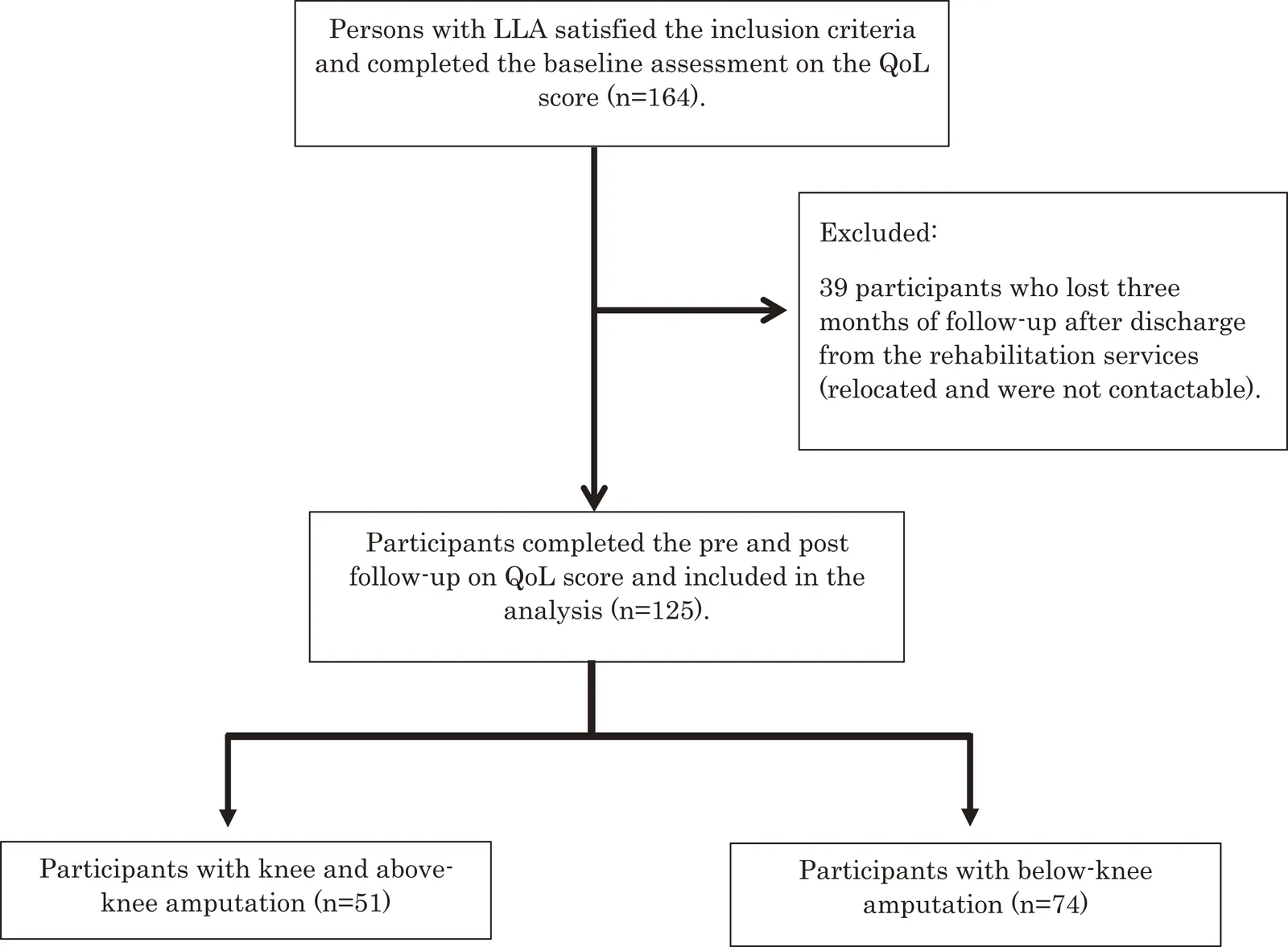
Flowchart of participant selection. LLA, lower limb amputation; QoL, quality of life

### Intervention

The PRCs provide comprehensive rehabilitation services (including physical therapy interventions and provision of prosthetics, orthotics, and other assistive devices) to persons with various disabilities, including amputations, and often offer dormitory facilities for inpatients^5,17)^.

Persons with LLA in Cambodia typically begin their rehabilitation journey at a PRC when they are referred from hospitals after their surgery^5)^. Their structured intervention process, which is the primary role of the physical therapist, encompasses several key stages^5)^ of the rehabilitation pathway^5)^:

1. Stump management and muscle strengthening: Initial focus is on wound healing, stump shaping, and strengthening the remaining limb muscles to prepare for prosthetic fitting.
2. Crutch or wheelchair training: Training in the effective use of assistive devices for mobility during the pre-prosthetic phase (with wheelchair training being for those with bilateral LLA).
3. Prosthetic fitting: To ensure the prosthetic fits well to the stump without pain or discomfort.
4. Gait training with the prosthetic limb: Once fitted with a prosthetic limb, persons with LLA undergo intensive gait training to relearn walking patterns and improve balance.
5. Navigating environmental challenges: Training extends to mastering functional mobility in various real-world environments, including walking on uneven surfaces and managing obstacles.

The standardized rehabilitation pathway as described above was followed with each of the 125 persons with LLA who fully participated in this study. This rehabilitation intervention lasted an average of 4 weeks, with the period between prosthesis fitting and commencing gait training lasting an average of 1 week. The study participants were followed up at 3 months after discharge from rehabilitation services, in compliance with the national standard procedure^5)^.

### Data collection

To promote disability inclusion and reduce data collection bias, 7 persons with disabilities who were not participating in the study were selected by each PRC as interviewers. Interviewers received training from the researcher in August 2013, which included training on data privacy and confidentiality, and an opportunity to practice administering the questionnaire.

A baseline questionnaire was administered to the 164 studied participants upon admission to the PRC, prior to the start of the rehabilitation intervention. Follow-up questionnaires were conducted with 125 participants 3 months after discharge from rehabilitation services at the routine follow-up visit. The baseline and follow-up questionnaires included the ComQOL-A5 questionnaire, developed by Professor Robert Cummins at Deakin University, Australia^18)^, and translated into the Cambodian language (Khmer)^10)^. Data regarding sociodemographic characteristics, health status, medical history, rehabilitation, and other support received were also collected.

The ComQOL-A5 is a multidimensional tool designed to evaluate both objective and subjective aspects of QoL across 7 domains: material well-being, health, productivity, intimacy, safety, community well-being, and emotional well-being^18)^. It captures both objective circumstances and subjective perceptions of importance and satisfaction within each domain, providing a nuanced understanding of a person’s priorities, perceived well-being, and QoL experience^10,18)^. The tool has been developed for use across diverse populations and cultures, demonstrating acceptable validity and reliability. It has been translated into Khmer for Cambodian use and has been used in previous QoL research for persons with disabilities (including LLA) and their caregivers in Cambodia^10,12)^. It provides a comprehensive framework for understanding the QoL of persons with disabilities, including persons with LLA, particularly in Cambodia, where socioeconomic factors and community integration are crucial^10,12)^.

### Statistical analysis

The data were entered into EpiData version 3.5 and then imported into STATA Version 18.0 (StataCorp, TX, USA) version 12 for analysis.

The study used a paired t-test to examine the difference between the QoL scores (mean of the overall objective and subjective scores) from baseline questionnaires before rehabilitation and follow-up questionnaires 3 months after discharge. Statistical significance level was set at p <0.05.

Variables with Yes/No responses were coded as Yes = 1 and No = 0, with No used as the reference category in all regression models. For example, the social support variable indicated whether participants received motivational, material, or financial support from family or friends. A positive regression coefficient represents an increase in QoL scores, whereas a negative coefficient indicates a decrease in QoL scores.

Univariate and multivariate regression analyses were conducted to identify factors associated with changes in QoL. The dependent variable was the change in QoL score, calculated as the difference between follow-up and baseline QoL measurements (follow-up score − baseline score).

First, univariate linear regression was employed to examine the association between each predictor variable (such as age, sex, marital status, number of children, educational level, employment history, level and cause of amputation, stump pain, phantom limb pain, time since amputation, use of assistive devices, comorbidities, and social support from family or friends [financial or material]) and the change in QoL score as the dependent variable. This preliminary analysis aimed to identify potential factors that might influence change in QoL score following rehabilitation.

Subsequently, a multivariable linear regression analysis was performed to evaluate the combined effects of multiple predictors while controlling for potential confounding factors. The multivariable model was constructed following an initial univariate screening process, in which variables with a p-value <0.25 were selected as candidate predictors. This liberal threshold was used because higher cut-off values help reduce the risk of excluding potentially important variables during early selection, as recommended in methodological literature^19)^.

To ensure the statistical validity of the findings, multicollinearity was assessed using variance inflation factors (VIFs). Pairwise correlations were also examined prior to regression modeling to identify highly correlated predictors. Correlation coefficients exceeding 0.80 were considered indicative of potentially overlapping information. A VIF value near 1 indicated no collinearity, whereas values above 5 suggested moderate concern and the potential need to remove the affected variable to enhance model stability and interpretability^20)^.

The predictor variables (cause of amputation, stump pain, and phantom limb pain) were included for the model of multivariate linear regression of the overall objective score of the QoL (Table 1), while the confounding factors (employment before amputation, time since amputation, stump pain, phantom limb pain, and relative/friend support) were included as covariates for the model of multivariate linear regression of the overall subjective score of the QoL (Table 2).

**Table 1.** Univariate and multivariate linear regression on overall average objective score analysis showing that the coefficient of factors correlates with the QoL

Variables	Univariate				Multivariate				
	Coef.	95% CI	p-Value	Overall p-value	Coef.	95% CI	p-Value	Overall p-value	VIF
Cause of amputation				0.019				0.054	2.70
Traffic accident (=2) vs. mine/UXO (=1)	−3.34	−11.05 to 4.36	0.391		−2.08	−9.66 to 5.50	0.588		5.72
Diabetes (=3) vs. mine/UXO (=1)	−6.99	−16.96 to 2.97	0.167		−4.87	−14.74 to 4.98	0.329		3.10
Peripheral vascular disease (=4) vs. mine/UXO (=1)	4.93	−5.36 to 15.22	0.344		5.73	−4.32 to 15.80	0.261		1.97
Cancer (=5) vs. mine/UXO (=1)	1.66	−9.61 to 12.94	0.770		1.66	−9.33 to 12.66	0.764		1.15
Accident (=6) vs. mine/UXO (=1))	−9.52	−18.83 to −0.21	0.045		−6.95	−16.26 to 2.34	0.141		1.50
Others (=9) vs. mine/UXO (=1)	−7.77	−16.42 to 0.87	0.078		−6.77	−15.25 to 1.69	0.116		3.13
Stump pain				0.002				0.002	1.28
Yes (=1) vs. No (=0)	−6.87	−11.26 to −2.49	0.002		−6.87	−11.26 to −2.49	0.002		1.28
Phantom limb pain				0.131				0.068	1.39
Yes (=1) vs. No (=0)	−5.76	−9.64 to 1.27	0.131		−3.88	−8.06 to 0.29	0.068		1.39
Other variables**									

**Other variables: Age group, sex, marital status, level of education, employment before amputation, comorbidity, time since amputation, level of amputation, history of receiving rehab services, and relative/friend support were analyzed via univariate linear regression. Each variable has p ≥0.25.

The multiple regression model significantly predicted the outcome variable (overall objective QoL change), *F*(8, 116) = 2.76, p = 0.008.

QoL, quality of life; Coef., coefficient; CI, confidence interval; VIF, variance inflation factor, UXO, unexploded ordnance

**Table 2.** Univariate and multivariate linear regression on overall average subjective score analysis showing that the coefficient of factors correlates with the QoL

Variables	Univariate				Multivariate				
	Coef.	95% CI	p-Value	Overall p-value	Coef.	95% CI	p-Value	Overall p-value	VIF
Employment before amputation				0.078				0.089	1.20
Yes (=1) vs. No (=0)	−4.94	−10.45 to 0.57	0.078		−4.75	−10.24 to 0.73	0.089		1.20
Time since amputation				0.024				0.023	2.34
>6–12 months (=2) vs. <6 months (=1)	−4.08	−10.59 to 2.41	0.216		−4.42	−10.85 to 1.99	0.175		5.25
>12 months (=3) vs. <6 months (=1)	−7.64	−14.64 to −0.65	0.032		−7.46	−14.37 to −0.56	0.034		4.45
Stump pain				0.133				0.167	3.21
Yes (=1) vs. No (=0)	−4.89	−11.31 to 1.51	0.133		−4.40	−10.68 to 1.87	0.167		1.23
Phantom limb pain				0.131				0.251	1.24
Yes (=1) vs. No (=0)	−4.18	−9.64 to 1.27	0.131		−3.26	−8.87 to 2.34	0.251		1.24
Relative/friend support				0.041				0.04	1.08
Yes (=1) vs. No (=0)	−7.76	−15.22 to −0.31	0.041		−7.70	−15.03 to −0.38	0.04		1.08
Other variable**									

**Other variables: Age group, sex, marital status, level of education, comorbidity, cause of amputation, level of amputation, and history of receiving rehab services were analyzed via univariate linear regression. Each variable has p ≥0.25.

The multiple regression model significantly predicted the outcome variable (overall subjective QoL change), *F*(8, 116) = 3.59, p <0.001.

QoL, quality of life; Coef., coefficient; CI, confidence interval; VIF, variance inflation factor

### Ethical consideration

This study was approved by the directors of each of the 7 PRCs and the National Ethics Committee for Health Research (NECHR) at the Cambodian Ministry of Health (issued approval number 0097 NECHR). Participants were verbally informed about data privacy and confidentiality by the trained data collectors. Informed consent was obtained by reading the consent information verbally to participants to ensure their understanding before signing the consent form.

## Results

### Sociodemographic characteristics

Table 3 presents the sociodemographic characteristics, baseline clinical conditions, and services from the pre-rehabilitation survey on admission to the PRC.

**Table 3.** Sociodemographic characteristics, baseline clinical conditions, and service utilization among participants who completed pre–post data collection and those lost to follow-up

Characteristics	Lost to follow-up (N = 39)		Completed pre–post data collection (N = 125)	
	n	%	n	%
Mean age (years)	43.89 (±18.27)		38.62 (±15.82)	
Sex				
Male	34	87.18	96	76.80
Female	5	12.82	29	23.20
Education				
No school	7	17.95	17	13.60
Primary school	15	38.46	61	48.80
Secondary school	8	20.51	33	26.40
High school	7	17.95	9	7.20
University	2	5.13	5	4.00
Marital status				
Single	14	35.90	34	27.20
Married	20	51.28	80	64.00
Widow/widower	5	12.82	11	8.80
Employment before amputation				
Yes	22	56.41	45	36.00
No	17	43.59	80	64.00
Breadwinner				
Self	12	30.77	14	11.20
Spouse	8	20.51	58	46.40
Children	4	10.26	15	12.00
Parents	12	30.77	32	25.60
Other relatives	3	7.69	6	4.80
Children				
Mean number of children	4.04 (±2.54)		3.01 (±2.28)	
Mean number of children under current supervision	1.78 (±1.56)		1.87 (±1.50)	
Additional support from relatives/ friends				
Yes	4	10.26	18	14.40
No	35	89.74	107	85.60
Level of amputation				
Knee and above	18	46.15	51	40.80
Below knee and ankle	21	53.85	73	58.40
Bilateral leg amputation	0	0	1	0.80
Duration of amputation				
Less than 6 months	21	53.85	74	59.20
More than 6 months to 12 months	11	28.20	28	22.40
More than 12 months	7	17.95	23	18.40
Cause of amputation				
Mine/UXO	7	17.95	7	5.60
Traffic accident	18	46.15	65	52.00
Other peripheral vascular diseases	6	15.38	17	13.60
Cancer	1	2.56	5	4.00
Other Accidents	1	2.56	12	9.60
Others	6	15.39	19	15.20
Stump pain				
Yes	2	5.13	47	37.60
No	37	94.87	78	62.40
Phantom limb pain				
Yes	13	33.33	47	37.60
No	26	66.67	78	62.40
Rehabilitation services				
No	31	79.49	94	75.20
Yes	8	20.51	31	24.80

N/n, number; UXO, unexploded ordnance

Of the 39 persons with LLA who missed the 3-month post-follow-up in the study, 12.82% (5) were females, and 51.28% (20) were married. The mean age was 43.89 years, and the average number of children was 4.04. Regarding education, 38.46% (15) had attended primary school. Before amputation, 43.59% (17) were unemployed. Upon admission to the PRC after amputation, 30.77% (12) relied on financial support from their parents, and 89.74% (35) reported no additional support from relatives or friends. Among the 39 amputees, most had below-knee amputations 53.85% (21) and had undergone amputation within the past 6 months 53.85% (21). Traffic accidents were the leading cause 46.15% (18), followed by mine/UXO incidents 17.95% (7). Stump pain was rare 5.13% (2), while one-third experienced phantom limb pain 33.33% (13). Access to rehabilitation services from other hospitals or organizations remained limited, with only 20.51% (8) having received such services.

Of the 125 persons with LLA who fully participated in the study, 23.20% (29) were females, and 64.00% (80) were married. The mean age was 38.62 years, and the average number of children was 3.01. Regarding education, 48.80% (61) had attended primary school. Before amputation, 64.00% (80) were unemployed. Upon admission to the PRC after amputation, 46.40% (58) relied on financial support from their spouses, and 85.60% (107) reported no additional support from relatives or friends.

In relation to their surgery, 58.40% (73) had undergone either below-knee or through-knee amputations, and 99.20% (124) had unilateral amputations, with 52.00% (65) of all cases caused by road accidents. The majority, 59.20% (74), had undergone amputation surgery within 6 months prior to accessing the PRCs.

Regarding pain, 37.60% (47) experienced stump pain, while 37.60% (47) reported phantom limb pain. Additionally, 24.80% (31) of participants received rehabilitation services from other hospitals or organizations, including physical therapy (32%), wheelchair provision (13%), and crutches (90%).

### Change in score of objective and subjective measurement for QoL

On admission to the physical rehabilitation program, the average overall objective QoL score was 48.10, and at follow-up (3 months after discharge from rehabilitation services), the average score was 62.03. This difference was statistically significant (p <0.001). Significant differences were also found in each of the defined domains of objective score measurement before and after rehabilitation (Table 4).

**Table 4.** Means of overall objective and subjective scores by domains

Variables	Overall subjective mean score			Overall objective mean score		
	Before services	After services	p-Value	Before services	After services	p-Value
Overall objective (7 domains)	48.10 (±9.32)	62.03 (±7.70)	<0.000	63.71 (±14.92)	77.59 (±10.16)	<0.000
Domains						
Well-being	40.79 (±20.10)	48.85 (±18.86)	<0.001	61.62 (±20.47)	77.95 (±17.74)	<0.001
Health	77.40 (±8.84)	91.93 (±8.05)	<0.001	69.93 (±17.17)	79.85 (±18.25)	<0.001
Productivity	43.85 (±17.59)	58.26 (±17.45)	<0.001	61.26 (±20.07)	72.84 (±19.84)	<0.001
Intimacy	55.44 (±21.28)	72.51 (±15.10)	<0.001	69.20 (±17.59)	81.51 (±13.25)	<0.001
Safety	43.85 (±16.93)	61.41 (±14.26)	<0.001	57.24 (±21.15)	76.88 (±15.79)	<0.001
Place in community	23.99 (±13.21)	33.40 (±11.90)	<0.001	57.24 (±15.35)	69.36 (±15.02)	<0.001
Emotional well-being	48.42 (±15.90)	65.16 (±13.96)	<0.001	70.12 (±22.39)	86.00 (±15.52)	<0.001

Similarly, the average overall QoL subjective score on admission to the PRC was 63.71, and then at follow-up, it was 77.59, a statistically significant difference (p <0.001). There were also significant differences in each of the defined domains of subjective score measurement when comparing scores at baseline with scores 3 months after discharge from rehabilitation services (Table 4).

Model fit: The multiple regression model significantly predicted the outcome variable (overall objective QoL change) *F*(8, 116) = 2.76, p = 0.008 (Table 1).

The multiple regression model significantly predicted the outcome variable (overall subjective QoL change), *F*(8, 116) = 3.59, p <0.001 (Table 2).

### Factors related to the change in the QoL score

In total, 13 variables were assessed using univariate and multivariate linear regression analysis for the overall average scores of both the objective and subjective QoL measurement. The results relating to the objective QoL scores are shown in Table 1 (univariate and multivariate linear regression on overall objective score), and results for the subjective QoL scores in Table 2 (univariate and multivariate linear regression on overall subjective score).

The results of the univariate linear regression analysis showed that both an absence of stump pain and the cause of amputation were significantly correlated with the increase in the overall average objective QoL score (Table 1), while the remaining 11 variables showed no significant association. Table 2 shows a significant relationship between a shorter time period from amputation to starting rehabilitation (specifically less than 6 months) and an increase in the overall average subjective QoL score, while the other 11 variables showed no significant association. Receiving social support before rehabilitation showed a significant adverse correlation with the increase in the overall average subjective QoL score.

The multivariate linear regression demonstrated that the presence of residual stump pain was associated with a lower overall average objective QoL score (β = −6.87; 95% confidence interval [CI] −11.26 to −2.49; p = 0.002) (Table 1). The multivariate linear regression also showed that participants who did not receive support from relatives or friends before rehabilitation had significantly higher overall average subjective QoL scores (β = −7.70; 95% CI −15.03 to −0.38; p = 0.040) (Table 2). In addition, the absence of residual stump pain was significantly associated with higher QoL scores (p = 0.002). In contrast, a longer delay (>12 months) between amputation and receipt of rehabilitation services was associated with a smaller improvement in subjective QoL (p = 0.023) (Table 2).

## Discussion

This study showed a significant increase in both overall average objective and subjective QoL scores among persons with LLAs 3 months after completing rehabilitation services at 7 PRCs in Cambodia, compared to baseline QoL scores at the time of admission to the PRCs. In the absence of a control group, this finding does not provide definitive evidence that receiving rehabilitation services results in an improvement in the QoL of persons with LLA, as the effects of other factors, including the passage of time since amputation, were not taken into account in the research design. However, the observed increase in QoL scores after rehabilitation is consistent with the findings of previous research, and the analysis offers valuable insights for the delivery of rehabilitation services, particularly in a developing country setting^21–23)^.

A cross-sectional study conducted in Cambodia in 2012 using the same QoL questionnaire found increased QoL for persons with LLA after rehabilitation^16)^. Other prospective studies among persons with LLA have also observed improvements in QoL after rehabilitation, though these were measured using a variety of different assessment instruments^21,24,25)^.

A study in Tunisia using the Short Form 36 quality-of-life score (SF-36) to measure the QoL at the first consultation and again at 12 months found significant improvement in the QoL of persons with LLA^24)^. A study in the Netherlands using the Dutch RAND-36 instrument observed improvements 6 months after rehabilitation compared to pre-rehabilitation across 9 domains, which included physical function, physical role limitations, social function, emotional role limitations, mental health, pain, vitality, general health, and perceived change in health^25)^. A prospective study undertaken in Canada using the Subjective Quality of Life Profile tool, which assesses physical and mental health, relationships, social roles, income, and spiritual life, also found significant improvements in QoL 3 months following rehabilitation^21)^.

It was noted that the causes of amputation and the age groups of participants in the studies in Cambodia were different from those in the aforementioned studies in developed countries. The most common reason for amputation in the studies in developed countries was diabetes^26)^, with participants being elderly adults, whereas in studies in Cambodia, the most common cause was road accidents in a younger adult group^7,27)^. It is therefore recommended that future studies should consider the cause of amputation and age group as predictor variables, as age has been found to be an important predictor of the QoL of persons with LLA^13)^.

In this study, participants with residual stump pain showed lower levels of increase in their objective QoL score compared to those without. Similar findings have been observed in a review study and other cross-sectional studies in the Netherlands and India^1,13,22)^. This suggests that the treatment of residual stump pain should be prioritized when the prosthesis is prescribed^28)^.

This study found that a longer delay between amputation and starting rehabilitation is adversely associated with QoL, specifically a delay of more than 12 months. This is supported by findings from a systematic review that observed greater improvement in walking ability following a shorter interval between surgery and rehabilitation admission^13,21,23)^, and a recent cross-sectional study in Brazil, which found a shorter time between amputation and starting rehabilitation to be a predictor of a higher perceived QoL (using a 36-item short-form questionnaire)^29)^. Therefore, it is recommended that professional rehabilitation services be provided to persons with LLA as early as possible after amputation^30)^.

An unexpected finding from this study was that support received from relatives or friends, whether motivational, material, or financial, was negatively associated with the increase in subjective QoL scores of persons with LLA.

Many studies have demonstrated that family and social support are significantly and positively associated with the QoL of persons with LLA. For example, Sinha et al. and Asano et al. reported that social and family support are important predictors of improved QoL among persons with LLA^13,31)^. Similarly, William et al. found that the degree of social support was a significant predictor of post-amputation outcomes, including pain interference, life satisfaction, and mobility levels^32)^. More recent evidence from a 2022 correlational study conducted in Iraq also identified a strong positive correlation between social support and QoL among 150 amputees, with participants who received higher levels of support reporting substantially better QoL outcomes^33)^.

However, qualitative findings highlight a more nuanced understanding of this relationship. A study by Abouammoh et al.^34)^ in Saudi Arabia underscored the complex, and at times contradictory, role of family and community support in the psychosocial adjustment of persons with LLA. Although such support is often considered beneficial, the authors found that certain forms, particularly those characterized by overprotection, excessive emotional involvement, or pity-driven behaviors, can inadvertently impede adjustment and negatively influence overall QoL^34)^.

Although this study found a negative association between support from relatives or friends and increases in subjective QoL, this result should be interpreted cautiously due to the single-group observational design. LLAs receiving more support at baseline may have had greater disability severity, psychosocial distress, or socioeconomic disadvantage, factors strongly associated with both an increased need for assistance and lower QoL. Such confounding by severity or social disadvantage is well documented in amputation research, where social support is typically higher among individuals with greater functional limitations or lower socioeconomic status. For example, studies from Iraq and the United States consistently show that amputees with lower income or poorer functional status report different levels of social support and QoL, indicating that these characteristics can strongly influence observed associations. Therefore, the negative association observed in this study may reflect underlying baseline differences rather than a true adverse effect of social support^33)^.

Reverse causation and measurement limitations may also explain the unexpected result. The support variable in this study captured only whether support was received, not its **quality, timing, intensity, or type**, all of which have been shown to influence QoL differently. Individuals with lower well-being at baseline may have sought more support, creating the appearance of a negative relationship over time. In addition, the measure did not differentiate empowering forms of support from support that may inadvertently foster dependence, which can affect rehabilitation outcomes^35)^.

Although psychological support data were not collected in this study, existing literature and clinical observations consistently demonstrate that LLA has profound effects on both physical and psychological well-being^36,37)^. These impacts often lead to significant changes in various aspects of life, including mobility, social participation, and emotional health. A recent study on persons with LLA in Cambodia reported that all participants (100%, n = 347) experienced psychological distress, which had a detrimental impact on their QoL. Furthermore, higher levels of psychological distress were significantly associated with reduced mobility^38)^. These findings underscore the importance of integrating mental health interventions, including peer support programs, into rehabilitation services for persons with LLA^37,38)^.

In our study, we observed that participants with phantom limb pain showed a lower increase in QoL, although this was not statistically significant. However, many studies have found phantom limb pain to be a significant predictor that adversely impacts the QoL of persons with LLA^1,13,22,39)^. Other studies have highlighted the importance of providing repeated and ongoing physiotherapy following rehabilitation in the successful treatment of phantom limb pain^13,39,40)^.

### Limitations

Although the subjective model explained a moderate proportion of the variance (R^2^ = 0.482, p <0.001), the objective model explained substantially less variance (R^2^ = 0.322) and was only weakly supported by the overall model significance (p = 0.008).

This likely reflects unmeasured determinants (e.g., baseline functional status and mobility, prosthesis characteristics and satisfaction, psychological distress, and social context). Future research should incorporate these covariates and use longer follow-up to enhance model fit and replicability.

This study used a single-group pre–post design rather than a randomized controlled trial (RCT), so it cannot be determined whether the observed increase in QoL was influenced by factors such as natural recovery, progression of time since the amputation, regression to the mean, other concurrent care, or other environmental factors, rather than rehabilitation alone.

Physical function and the ability to walk using a prosthetic limb were not reported in this research^11,41)^. For young persons with traumatic amputations in Cambodia, the ability to walk and return to work may be significantly associated with QoL. Follow-up was only short-term, at 3 months after rehabilitation discharge. The residual limb will continue to mature and change shape for at least 6–9 months after amputation surgery, and persons with LLA will continue to make functional gains after 3 months. Therefore, longitudinal follow-up at regular intervals is warranted. Prosthetic components for participants were not reported in this article; these may potentially influence QoL and functional outcomes. Lack of information on functional outcomes is a limitation of this study. Not measuring psychological status may have influenced our interpretation of the results, as some studies have found that depression is associated with poor QoL in persons with LLA^13,24,37,38)^.

## Conclusions

To conclude, while this single-group pre–post study cannot establish causality, the observed increase in QoL scores after the rehabilitation intervention highlights the practical importance of PRC services for persons with LLA. The study suggests that these benefits may be maximized by initiating rehabilitation as soon as possible after amputation to enhance its potential impact on QoL, as delays, particularly those exceeding 12 months, were significantly associated with poorer QoL outcomes.

Although pain management and social support are associated with changes in QoL among persons with LLA, their causal impact remains unproven. Future research should include longer prospective longitudinal studies to understand how QoL can be sustained after rehabilitation, as well as RCTs to determine the causal relationships between rehabilitation and improved QoL for persons with LLA, and to assess whether targeted interventions in pain management and social support effectively improve outcomes.
